# Supplementary material for: Automatic and feature-specific prediction-related neural activity in the human auditory system
Source: Nat Commun. 2019 Aug 1;10:3440. doi: 10.1038/s41467-019-11440-1 (PMC6672009; doi:10.1038/s41467-019-11440-1)
Supplement: Supplementary file 2 — Reporting Summary [file 41467_2019_11440_MOESM2_ESM.pdf]

natureresearch

Corresponding author(s): Gianpaolo Demarchi

Last updated by author(s): Jul 4, 2019

Reporting Summary

Nature Research wishes to improve the reproducibility of the work that we publish. This form provides structure for consistency and transparency in reporting. For further information on Nature Research policies, see Authors & Referees and the Editorial Policy Checklist.

Statistics

For all statistical analyses, confirm that the following items are present in the figure legend, table legend, main text, or Methods section.

n/a	Confirmed	
☐	☒	The exact sample size (*n*) for each experimental group/condition, given as a discrete number and unit of measurement
☐	☒	A statement on whether measurements were taken from distinct samples or whether the same sample was measured repeatedly
☐	☒	The statistical test(s) used AND whether they are one- or two-sided<br>*Only common tests should be described solely by name; describe more complex techniques in the Methods section.*
☐	☒	A description of all covariates tested
☐	☒	A description of any assumptions or corrections, such as tests of normality and adjustment for multiple comparisons
☐	☒	A full description of the statistical parameters including central tendency (e.g. means) or other basic estimates (e.g. regression coefficient) AND variation (e.g. standard deviation) or associated estimates of uncertainty (e.g. confidence intervals)
☐	☒	For null hypothesis testing, the test statistic (e.g. *F*, *t*, *r*) with confidence intervals, effect sizes, degrees of freedom and *P* value noted<br>*Give P values as exact values whenever suitable.*
☒	☐	For Bayesian analysis, information on the choice of priors and Markov chain Monte Carlo settings
☒	☐	For hierarchical and complex designs, identification of the appropriate level for tests and full reporting of outcomes
☒	☐	Estimates of effect sizes (e.g. Cohen's *d*, Pearson's *r*), indicating how they were calculated

Our web collection on statistics for biologists contains articles on many of the points above.

Software and code

Policy information about availability of computer code

Data collection	Elekta standard acquisition sw
Data analysis	MATLAB, (MathWorks, Natick NA), https://www.mathworks.com/products/matlab.html<br>Fieldtrip toolbox, (Oostenveld et al., 2011), http://www.fieldtriptoolbox.org/<br>CoSMoMVPA toolbox, (Oosterhof et al., 2016), http://www.cosmomvpa.org/<br>Psychophysics Toolbox, (Brainard 1997), http://psychtoolbox.org/<br>Data analysis pipeline is available at the corresponding author's github repository (https://github.com//gdemarchi)

For manuscripts utilizing custom algorithms or software that are central to the research but not yet described in published literature, software must be made available to editors/reviewers. We strongly encourage code deposition in a community repository (e.g. GitHub). See the Nature Research guidelines for submitting code & software for further information.

Data

Policy information about availability of data

All manuscripts must include a data availability statement. This statement should provide the following information, where applicable:

- Accession codes, unique identifiers, or web links for publicly available datasets
- A list of figures that have associated raw data
- A description of any restrictions on data availability

Yes. In the Data and Code Availability section is stated. The raw was not (yet) put online for a mere problem of space and bandwidth. The raw MEG data amounts at about 300 G. We put on Zenodo (DOI: 10.5281/zenodo.3268713) a downsampled version of the raw data. We can provide upon reasonable request the raw data, as well as intermediate stages data, plus the scripts to generate them.

Field-specific reporting

Please select the one below that is the best fit for your research. If you are not sure, read the appropriate sections before making your selection.

☒ Life sciences ☐ Behavioural & social sciences ☐ Ecological, evolutionary & environmental sciences

For a reference copy of the document with all sections, see nature.com/documents/nr-reporting-summary-flat.pdf

Life sciences study design

All studies must disclose on these points even when the disclosure is negative.

Sample size	No statistical methods were used to pre-determine sample sizes but we chose the sample size based on literatures in the field.
Data exclusions	No subject were excluded from the initial analysis. One subject was discarded after realizing the s/he received the wrong stimulation sequence. No bad trials were excluded, and bad channels were fixed/interpolated by MaxFilter®. More details in the Methods section.
Replication	Indeed we are self replicating this study within our group, keeping most of stimulation parameters, and either changing the stimulation rate, or manipulating the attention of the subjects. The results of the other studies, at least with the parts in common with this one, essentially coincide.
Randomization	We had only one group of healthy adult subjects. Our subjects were randomly recruited from the Salzburg student population, plus few non (no more/ex student) external subjects.
Blinding	Not applicable

Reporting for specific materials, systems and methods

We require information from authors about some types of materials, experimental systems and methods used in many studies. Here, indicate whether each material, system or method listed is relevant to your study. If you are not sure if a list item applies to your research, read the appropriate section before selecting a response.

Materials & experimental systems

n/a	Involved in the study	
☒	☐	Antibodies
☒	☐	Eukaryotic cell lines
☒	☐	Palaeontology
☒	☐	Animals and other organisms
☐	☒	Human research participants
☒	☐	Clinical data

Methods

n/a	Involved in the study	
☒	☐	ChIP-seq
☒	☐	Flow cytometry
☐	☒	MRI-based neuroimaging

Human research participants

Policy information about studies involving human research participants

Population characteristics	The subjects were all healthy, draw from the local population of university students. Few subjects were older (senior phds, postdocs, technical assistants and researchers)
Recruitment	They were recruited in the usual ways, that are: mailing lists, facebook, word of mouth, physical bulletin boards.
Ethics oversight	The Ethical Committee of the University of Salzburg

Note that full information on the approval of the study protocol must also be provided in the manuscript.

Magnetic resonance imaging

Experimental design

Design type	We only employed anatomical MR images to coregister the functional MEG data onto a brain iin roughly half of the subjects. For the other half a template was employed and morphed using the head shape points
Design specifications	N.A.
Behavioral performance measures	N.A.

Acquisition

Imaging type(s)	Structural
Field strength	1.5-4
Sequence & imaging parameters	The anatomical MR images were collected with different scanners of different manufacturer, so the parameters are different
Area of acquisition	Whole brain

Diffusion MRI ☐ Used ☒ Not used

Preprocessing

Preprocessing software	N.A.
Normalization	N.A.
Normalization template	N.A.
Noise and artifact removal	N.A.
Volume censoring	N.A.

Statistical modeling & inference

Model type and settings	N.A.
Effect(s) tested	N.A.

Specify type of analysis: ☒ Whole brain ☐ ROI-based ☐ Both

Statistic type for inference (See Eklund et al. 2016)	N.A.
Correction	N.A.

Models & analysis

n/a	Involved in the study
☒	☐ Functional and/or effective connectivity
☒	☐ Graph analysis
☒	☐ Multivariate modeling or predictive analysis
